# Dr. Marshall on the Cholera in Glasgow

**Published:** 1832-01-01

**Authors:** 


### CHOLERA AT PORT-GLASGOW. By *John Marshall*, M.D. 8vo. pp. 80, 1831.

PARLIAMENTARY reform has tended to check the cholera-phobia in this country, during the last four or five months; but still the terror broke out occasionally, and attracted, from time to time, the attention of the public. The steady approach of the enemy will probably re-excite the panic, especially during the prorogation of Parliament, when the political mania may experience a remission of its violence. Notwithstanding the regular advances which this terrible malady has hitherto made, from East to West, we continue to indulge our hopes and our expectations that the disease, if it reach our shores, will come in a mitigated form, or at all events, be met by more stubborn subjects, and a "tougher opposition," than it has encountered on the other side of LA MANCHE.

The author of the pamphlet before us informs us that he did not seek publicity, in the present case, but that it was thrust upon him, as the Bard of Avon says is sometimes the case with honours of a different kind. Having attended some cases of cholera in Glasgow, which appeared to be different from the ordinary disease of this country, he felt it to be his duty to communicate the results of his observations to the Privy Council—and these communications were thought to be of so much importance, that a medical officer was dispatched to the North to investigate the subject. Drs. Daun and Badinach were the examinators, on this occasion; and it appears that the former of these gentlemen reported to the Privy Council, that the cases in question were not the cholera of India, but the disease of this country. Some attacks were made on Dr. Marshall in one of the Glasgow papers, insinuating that he had caused needless alarm, and that he shewed some ignorance or inattention in the matter. To free himself from what he considered ungenerous and unjust aspersions on his professional character, Dr. Marshall has thought it necessary to publish the cases, in order that the profession might be able to judge whether or not he had just cause for alarm, and therefore good grounds for communicating with the Privy Council. We shall not enter into the preliminary discussion of the author respecting the identity or non-identity of the cholera of India and that of this country. We shall give the first case, as it is that which made such a noise in the newspapers,—the only one that proved fatal—and probably was the cause of the dispatch to the Privy Council.

*Case. July 2d,* 1831. " JOHN MURRAY, ætat. 25, reported to be a healthy and remarkably temperate man, rather of a slender make and pale complexion, labourer in a sugar-house here; became rather unwell last evening, went to bed early, and about one this morning was seized with purging, accompanied with severe pain of bowels, soon after with retching and vomiting, quickly followed by spasm, or as it is called by the patient, 'cramps' of the whole body and limbs, excessive sickness, shivering, and cold perspiration, with coldness of the extremities; what was the nature of these first evacuations could not be ascertained, as he had during the night retired from the house when urged by the calls of nature.

He is reported to have continued in this state till some time in the forenoon, when the purging ceased, and an emetic was ignorantly administered, after the operation of which, he is described to have become worse.

At nine, p.m. I saw him for the first time, and found him labouring under the following symptoms:—retching, instant vomiting of every thing swallowed, excruciating pain of abdomen, not increased by pressure. Has recently evacuated by the bowels matter reported to have 'resembled half-boiled white of egg.' Spasm of the whole body and limbs coming on in paroxysms, of which I witnessed several—sense of intolerable heat over the region of the stomach, urgent thirst, pulse almost imperceptible at the wrists, quite so at the temples, surface of the body cold and clammy, skin of a blueish or leaden hue, features collapsed, eyeballs sunk in their sockets, remarkable lividity of countenance.

Warm brandy and water were ordered to be freely given, and five grains of the Pil. Thebaicæ, E. P. every hour while the vomiting and spasms continued. Warm flannels, and bottles with warm water to be applied to the body and extremities, with a sinapism over the stomach.

3d—The patient has had a restless night, yet, upon the whole, there is a perceptible amelioration of the urgent symptoms. The intervals of retching and spasms are longer than yesterday, thirst unabated, but what is swallowed less immediately rejected, pulse quick and more distinct at the wrists, heat of body increased, but requiring constant hot applications to keep it up. Has had no stool to-day. Has taken eight of the pills above ordered, containing in all four grains of opium.

Continue hot gruel with brandy, repeat the sinapism. To have one grain of opium, and three grains of calomel every three hours.

4th—Considerable re-action has taken place, spasms nearly ceased, the stomach has retained a little gruel and brandy, no evacuation from the bowels, heat of skin more easily sustained, pulse stronger, countenance less ghastly. Owing to the extremely penurious habits of the patient, and the absence of all relations or responsible attendants, I find my prescriptions and directions very ill executed.

To have a lavement administered immediately, and repeated at intervals till the bowels are opened, and to continue the brandy gruel.

Eleven o'clock, p.m.—Considerably worse, constant singultus, extremities cold, spasms gone; two lavements have been administered, first was retained two hours, and came off with the second quite unaltered, said to have been mixed with a whitish matter, which having sunk to the bottom, was not observed till it was being thrown out. Had an evacuation from the bowels about an hour afterwards, of a whitish colour, unmixed with fæces.

Continue hot brandy and water. To have two compound opium pills immediately.

5th—Patient is in every respect worse, singultus constant, is reported to have had, since last report, involuntary evacuation of the bowels of a whitish matter. Died at 5 p.m. No urine had been voided for three days before death.

I never, to my knowledge, saw John Murray till called to him on the evening of the 2d, when it was quite impossible to judge what his personal appearance might have been. From the nature of the work which he performed in the sugar-house, I am certain, however, that he must have been a man of very considerable muscular strength, because no other would have been hired or retained in that particular department.

Much has been said and written about this unfortunate man having been at an 'Irish wedding,' and all his sufferings and the disease of which he died, have, humanely, been ascribed to his *supposed* excesses on that occasion. I have taken considerable trouble to obtain the most correct information possible on this subject; and by the most minute inquiries at the master and manager of the sugar-house, and also at his fellow-workmen, I learn that on Monday, 27th June, he was at the marriage of an Irishman, where he got intoxicated. That he was at his work as usual on the morning of Tuesday, 28th, and continued to perform it with all his accustomed ease and alacrity up to seven o'clock on the evening of Friday, 1st of July. Facts which must for ever set aside the fable of the 'Irish wedding.'

A week after this patient was buried, I was told that an emetic had been exhibited at one or two on the afternoon of July 2. In the course of the enquiries above alluded to, I have learned that one of his companions had procured, from a druggist, six grains of tartrate of antimony, with a printed direction that 'it should be dissolved in half a pint of water, a table-spoonful of this solution to be given every fifteen minutes, till vomiting takes place.'

It is averred by those who were present, that only a very small portion of it was swallowed, about two o'clock on *the morning* of July 2. But let us, for the sake of argument, allow that the whole was swallowed at one draught, could these six grains of emetic tartar have produced the morbid symptoms which I witnessed when called to him 19 hours after? We all know that this drug reduces the powers of life in a most extraordinary manner, subduing the outrageous maniac to the helplessness and docility of infancy, producing in such cases the sleep that had long been absent; and we also know, that in *over doses*, (such as that mentioned by Orfila, when an ounce was swallowed,) it produces symptoms highly analogous to those of cholera.* A fact not lost sight of by my *friend*, H. M.

I am ready to allow, that it may, in the present instance, have added to the debility under which the man laboured; but will any one venture to assert, that it produced deadly sickness, retching, cramp, constant inclination to go to stool, and floods of cold perspiration, *five hours before it was swallowed?*

In the presence of Dr. Daun, I, *for the first time*, learned from a medical practitioner here, that he had been called to Murray; had visited him before eight o'clock in the morning of July 2, and had prescribed a dose of castor oil *and an anodyne draught*, *because* he 'considered it to be a case of abdominal visceral inflammation.' But he returned no more to inquire into the progress of the 'inflammation,' under this very novel prescription.

To plead in his own defence that he absented himself 'because another was called in,' is quite in vain, since thirteen hours elapsed between the two circumstances. I observe by the Russian Reports, given in the Edinburgh Medical Journal for July, 1831, that similar mistakes were made by the Orenburg phy-

* "Some highly esteemed practitioners in India have advocated the use of antimonal emetics in Cholera."

sicians when Cholera first appeared there; but I do not observe that any of them left the miserable patients, without advice or assistance, to combat alone the fury and the agony of the disease." 17.

For our own parts, the perusal of the above case has left no other impression on our minds than that it was one of common—perhaps *severe* cholera Britannica, aggravated, nay rendered fatal, by the tartrite of antimony. The man may have taken only a part of the six grains; but a single grain of emetic tartar, exhibited in such a highly irritable state of the stomach, might very well make all the difference between death and recovery. Twenty-four cases are reported by Dr. Marshall, but only this one died—a strong proof that the disease was not of a very fatal character, however judicious (and we acknowledge Dr. Marshall's skill) might be the remedial measures. Calomel, opium, and the warm bath, were the chief measures employed in all these cases—and all, except Murray, recovered.

Dr. Marshall, in his concluding observations, quotes a statement from an aged medical friend of his, Dr. Carmichael, who had practised many years since in India, and which appears to us rather extraordinary. Dr. C. informed our author that, in the Indian cholera, he had *always* observed a "*copious black vomiting.*" Dr. Marshall says that this observation of his aged friend "exactly coincides with the account given by the earlier writers who have described the cholera of India." This does not tally with the accounts from Bontius and Curtis downwards—and we should be disposed to place little reliance on accounts previous to their time, in India at least.

"It has been a general, *and hitherto an undisputed remark, that Cholera in this country has its origin in the too free use of fruit, unripe or otherwise, and also of crude vegetable matter;* and we find it commonly appear about the middle of August, and prevail till the latter end of September.

In the present year it appeared six weeks earlier, and in no one case which I met with, had green vegetables been an article of diet at all. In only three cases had fruit been recently used, and in these cases it was very small in quantity." 72.

We are much surprised at the above remarks; for, in the whole course of our lives, we have hardly met with a practitioner, of the slightest talent or observation, who subscribed to the antiquated and ridiculous notion, that our autumnal cholera was owing to the eating of rife fruit. We might just as well argue, that Summer is owing to the swallows that flock to this country in that season.

We beg to state that we throw not the slightest blame on Dr. Marshall for his report to the Privy Council on the case of Murray—and that we think Dr. Henry Marshall's letter, in the Glasgow Herald, was too sarcastic on his namesake. From all that appears in the document before us, there is no foundation for accusing Dr. M. of propagating the idea, that—"the Russian cholera has arrived at Port-Glasgow on board a ship laden with flax from Riga." Cholera has been much more severe and much more prevalent this last Autumn, in these isles, than usual; and, considering the extent of the cholera-phobia in this country during the Summer, we do not wonder that Dr. Marshall was startled by the fatal case which first occurred to him. We apprehend, however, that the hot water in which he has got immersed by his conscientious report to the Privy Council, will somewhat cool his philanthropy, and render him more cautious, in future, how he addresses himself to courts and quarantines. Considering that every one now writes with a *steel* pen, there is more affinity between the sword and the " goose-quill" than in the days of Hudibras.

